# Evaluation of basophil allergen threshold sensitivity (CD-sens) to peanut and Ara h 8 in children IgE-sensitized to Ara h 8

**DOI:** 10.1186/s12948-014-0007-3

**Published:** 2015-04-15

**Authors:** Susanne Glaumann, Caroline Nilsson, S G O Johansson, Anna Asarnoj, Magnus Wickman, Magnus P Borres, Anna Nopp

**Affiliations:** Department of Clinical Science and Education, Södersjukhuset, Centre for Allergy Research, Karolinska Institutet, Stockholm, Sweden; Sachs’ Children and Youth Hospital, Södersjukhuset, 118 83 Stockholm, Sweden; Department of Medicine, Clinical Immunology and Allergy Unit, Karolinska Institutet and Hospital, Stockholm, Sweden; Institute of Environmental Medicine, Karolinska Institutet, Stockholm, Sweden; Department of Women’s and Children’s Health, Karolinska Institutet at Astrid Lindgren Children’s Hospital, Stockholm, Sweden; Thermo Fisher Scientific, Uppsala, Sweden; Department of Women’s and Children’s Health, Uppsala University, Uppsala, Sweden

**Keywords:** rAra h 8, Basophils, Birch pollen allergy, CD63, CD-sens, Children, Cross-reactivity, Flow cytometry, Peanut allergy, Pediatrics

## Abstract

**Background:**

Diagnosing peanut allergy properly is important and can be achieved by combining clinical history with various diagnostic methods such as IgE-antibody (IgE-ab) measurements, skin-prick test, basophil allergen threshold sensitivity (CD-sens) and food challenge. We aimed to evaluate CD-sens to peanut, Ara h 8 and Gly m 4 in relation to an oral peanut challenge in children IgE-sensitized to birch, peanut and Ara h 8 avoiding peanuts.

**Methods:**

Twenty children IgE-sensitized to birch pollen and Ara h 8, but not to Ara h 1, Ara h 2 or Ara h 3 were challenged orally with roasted peanuts. Blood samples were drawn for IgE-ab and CD-sens analysis. To measure CD-sens, basophils were stimulated *in vitro* with decreasing doses of allergens until threshold sensitivity was reached.

**Results:**

All children passed challenge without objective symptoms, but mild oral allergy syndrome (OAS) symptoms were reported in 6/20 children. Nineteen of twenty children were negative in CD-sens to peanut but 17/20 were positive to rAra h 8. Eleven of twenty children were positive in CD-sens to rGly m 4.

**Conclusion:**

Positive CD-sens to rAra h 8 show that the Ara h 8 IgE-ab sensitized basophils can be activated by a rAra h 8 allergen and initiate an allergic inflammation despite a negative challenge. Hence, children sensitized to Ara h 8 but not to peanut storage proteins may be at risk for systemic allergic reaction when eating larger amounts of peanuts but most likely don’t have to fear smaller amounts.

**Electronic supplementary material:**

The online version of this article (doi:10.1186/s12948-014-0007-3) contains supplementary material, which is available to authorized users.

## Background

Clinical reactions to peanut vary and the severity of the reaction is often hard to predict [1-3]. Since peanut allergy often is lifelong and affects quality of life a proper allergy diagnosis is important, but can be difficult to achieve [4]. The diagnosis is usually based on clinical history, skin-prick test and presence of IgE-antibodies (IgE-ab) in serum [4,5]. However, it often needs to be confirmed by an oral challenge. Today it is possible to investigate the IgE-ab pattern to individual peanut allergen components. Sensitization to Ara h 1, Ara h 2 and Ara h 3, the major peanut storage proteins, is associated with systemic allergic reactions [1,6-8]. IgE-ab to the lipid transfer protein (LTP) Ara h 9 could also cause systemic reactions to peanuts and is often seen in the Mediterranean area [9]. In Northern Europe IgE-ab to Ara h 8, a PR-10 protein, is common due to allergenic cross-reaction with the birch pollen allergen Bet v 1 [10]. However, it has recently been shown that children with a mono-sensitization to Ara h 8 usually tolerate peanuts without any severe allergic reactions [2]. Gly m 4 in soy is another PR-10 protein similar to that of Bet v 1 but in contrast to Ara h 8, sensitization to Gly m 4 has been reported to cause systemic reactions [11,12].

Basophils are important effector cells in IgE-mediated allergy [13] and by stimulating the basophils in vitro with decreasing doses of allergen, the smallest amount of allergen able to activate the basophils measured by CD63 expression is presented as basophil allergen threshold sensitivity (CD-sens). [14,15]. Analyses of CD-sens have shown promising results in predicting allergic reactions to both food and inhalant allergens [1,15-17].

The primary aim of the present study was to evaluate CD-sens to peanut and Ara h 8 in relation to an oral peanut challenge in children with IgE-ab to birch and rAra h 8, but not to rAra h 1, Ara h 2 and Ara h 3. A secondary aim was to evaluate CD-sens to rGly m 4 in the same group of children.

## Results

### Peanut challenge

Demographic data of the 20 children in the study are shown in Table 1. All children were challenged with 11.1 g of peanuts without any objective symptoms and no DBPCFC were performed. Of the seven children who reported symptoms after ingesting peanuts before the challenge (facial oedema, cough, mouth itch, perception of pharyngeal swelling and skin itch) two had OAS at the challenge. Six children experienced OAS but these symptoms subsided spontaneously without medication within one hour after last dose of peanut and were regarded as a negative peanut challenge.

**Table 1:** **Patient’s characteristics at inclusion**

**Number of patients**	
**n (%)**	20 (100)
**Male**	
**n (%)**	9 (45)
**Age, years**	
** Median (range)**	14.5 (5–-18)
**Co morbidity**	
Asthma, n (%)	14 (70)
Hay fever, n (%)	17 (85)
Food allergy other than peanuts and tree nuts, n (%)	15 (75)
Eczema, n (%)	10 (50)
**IgE-ab (kU**_**A**_ **/L) at inclusion**	
Birch, median (range)	52 (0.7- > 100*)
Peanut, median (range)	1.1 (0.1-8.5)
rAra h 8, median (range)	10.5 (0.5- > 100*)
**Peanut consumption before challenge**	
Reported symptoms after accidental intake of peanuts, n (%)	3 (15)
Never eaten peanuts**, n (%)	17 (85)

*IgE-ab levels >100 kUA/L measured in routine clinical diagnostic work-up before inclusion is described as 101 kUA/L.

**Claimed they have never eaten peanuts because of earlier information about IgE-ab sensitization to peanuts.

### IgE-antibodies

At the inclusion all children had IgE-ab to peanut and rAra h 8 > 0.35 kU_A_/L but no IgE-ab to rAra h 1, rAra h 2 and rAra h 3 (IgE-ab < 0.35 kU_A_/L). At the time of challenge, all children had still IgE-ab (>0.1 k_A_U/L) to peanut and the median (range) was 0.7 (0.1-16.1) kU_A_/L. The median for rAra h 8 was 6.4 (0.5-131.7) kU_A_/L and for rBet v 1 30.1 (1.5-202.6) kU_A_/L. Three children had low levels of IgE-ab to rAra h 2 (0.2-0.4 kU_A_/L). All children but one had IgE-ab to rGly m 4 with a median of 4.9 (1.5-18.9) kU_A_/L (Table 2). There was no significant difference in IgE-ab levels to peanut (p = 0.93) or rAra h 8 (p = 0.93) in children with or without OAS at the challenge.

**Table 2:** **Immunological analysis at challenge: CD-sens values, IgE-antibodies and OAS**

**Patient id**	**1** ^**#**^	**2**	**3**	**4**	**5**	**6**	**7**	**8**	**9**	**10**	**11**	**12**	**13** ^**#**^	**14** ^**#**^	**15**	**16** ^**#**^	**17** ^**#**^	**18** ^**#**^	**19** ^**#**^	**20**
**Symptoms**	0	0	0	0	OAS	OAS	OAS	0	0	0	OAS	0	OAS	0	0	0	OAS	0	0	0
**CD-sens peanut**	0	0	0	12.5	0	0	0	0	0	0	0	0	0	0	0	0	0	0	0	0
**CD-sens rAra h 8**	1.5	0	23.2	82.8	5.9	0	38.9	6.0	6.1	30.6	10.4	4.8	0	1.2	4.0	68.7	13.5	3.0	2.0	50
**CD-sens rGly m 4**	1.1	Positive	0	51.7	0	0	5.0	2.0	0	8.2	41.8	14.6	0	1.2	0	15.4	0	0	0	17
IgE*	359	2460	1030	169	119	1110	959	1670	253	439	428	2080	130	54.6	264	44	59	147	503	190
IgE-ab Peanut**	3.1	0.7	4.9	2.9	0.6	0.3	16.1	5.7	1.8	0.7	4.8	5.4	0.3	0.3	0.6	0.2	0.13	0.2	0.6	1.0
IgE-ab rAra h 8**	6.4	0.8	44.9	38.1	6.4	0.5	16.4	132	1.4	11.1	14.9	21.7	6.3	2.4	7.4	4.5	2.9	1.1	5.1	17.6
IgE-ab rAra h 1**	<0.1	0.1	<0.1	<0.1	<0.1	<0.1	0.1	<0.1	0	<0.1	<0.1	<0.1	<0.1	<0.1	<0.1	<0.1	<0.1	<0.1	<0.1	<0.1
IgE-ab rAra h 2**	0.1	0.2	0.1	0.4	<0.1	0.1	0.1	0.1	<0.1	<0.1	<0.1	0.3	0.1	<0.1	<0.1	<0.1	<0.1	<0.1	<0.1	<0.1
IgE-ab rAra h 3**	<0.1	0.1	<0.1	<0.1	<0.1	<0.1	0.1	<0.1	<0.1	<0.1	<0.1	0.1	0.1	<0.1	<0.1	<0.1	<0.1	<0.1	<0.1	<0.1
IgE-ab rAra h 9**	0.2	0.1	0.1	0	0	0.1	4.3	0.2	0	0	0	0.1	0	0	0	0.3	0	0	0	0
IgE-ab rGly m 4**	7.5	0.2	21.0	14.9	1.7	0	37.2	77.6	1.1	16.2	19.7	25.6	5.4	2.0	4.5	1.9	1.4	0.5	3.9	15.8
IgE-ab rBet v 1**	47.1	1.5	124	97.3	35.1	25.1	36.4	203	3.6	76.6	48.8	135	21.1	8.4	20.6	9.8	12.6	7.0	18.7	52.0
**IgE-ab fraction of rGly m 4**	2.1	0.01	2.1	8.8	1.4	0	3.9	4.6	0.4	3.7	4.6	1.2	4.1	3.6	1.7	4.4	2.3	0.4	0.8	8.3
**IgE-ab fraction of rAra h 8**	1.8	0.03	4.4	22.5	5.3	0.04	1.7	7.9	0.6	2.5	3.5	1.0	4.9	4.3	2.8	10.2	4.9	0.7	1.0	9.3
**IgE-ab fraction of Peanut**	0.9	0.03	0.5	1.7	0.5	0.03	1.7	0.3	0.7	0.2	1.1	0.3	0.2	0.6	0.2	0.5	0.2	0.1	0.1	0.5
**IgE-ab fraction of rBet v 1**	13.1	0.1	12.1	57.5	29.5	2.3	3.8	12.1	1.4	17.5	11.4	6.5	16.2	15.3	7.8	22.2	21.4	4.8	3.7	27.3

OAS = Oral Allergy Syndrome.

^#^symptoms reported before challenge; *kU/L; **kU_A_/L.

### CD-sens

**Figure 1:**
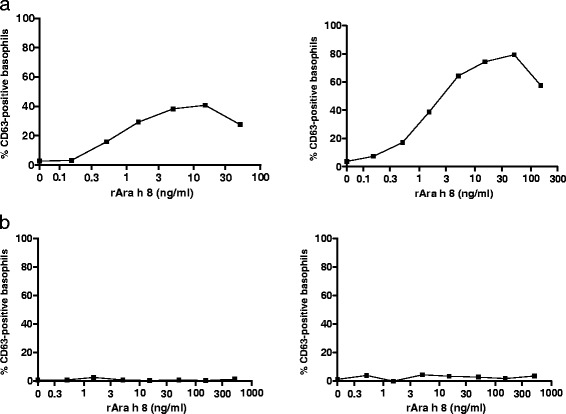
**a Cytometric results of two patients with positive CD-sens to Ara h 8: b Cytometric results of two patients with negative CD-sens to Ara h 8.**

All children but one (Patient 4) were negative in CD-sens to peanut. This child had at the time of challenge, but not at inclusion, IgE-ab to rAra h 2 (0.4 kU_A_/L) (Table 2). Seventeen children (85%) were positive in CD-sens to rAra h 8. At the time of the challenge the median of CD-sens to rAra h 8 was 5.9 (0–82.8). Levels of Ara h 8 IgE-ab in the three children with negative CD-sens to rAra h 8 were 0.5, 0.8 and 6.3 kU_A_/L and the corresponding IgE-ab fraction size to rAra h 8 was 0.03%, 0.04% and 4.9%, respectively (Table 2). Figure 1 is showing the flow cytometric results of representative cases with positive and negative CD-sens results. Eleven children (55%) were positive in CD-sens to rGly m 4 and the median was 1.1 (0–51.7). One child was positive in CD-sens to rGly m 4, but the CD63 expression barely reached the cut-off; hence no numerical CD-sens value could be calculated (Table 2).

### CD-sens and OAS

No significant difference in CD-sens to rAra h 8 was found between children with (n = 6) and without (n = 14) OAS at the peanut challenge.

## Discussion

We have investigated basophil allergen threshold sensitivity, CD-sens, to peanut, rAra h 8 and rGly m 4 in 20 children with a suspected peanut allergy. All children had IgE-ab to peanut and rAra h 8 but not to rAra h 1, rAra h 2 or rAra h 3 at the inclusion. An oral peanut challenge was negative in all children. However, 85% (n = 17) of the children were positive in CD-sens to rAra h 8, indicating that they had Ara h 8 IgE-ab sensitized basophils which could be activated by an intact rAra h 8 protein and initiate an allergic inflammation.

The diagnosis of peanut allergy is usually based on case history, presence of IgE-ab and a positive SPT. In children with food allergy it is not uncommon to have IgE-ab to different foods despite not having clinical symptoms after exposure. On an individual basis, the levels of IgE-ab or SPT wheal size to peanut, can neither predict an allergic reaction nor its severity. The probability for a reaction increases with elevated levels of peanut IgE-ab [18]. However, in Northern Europe where IgE-ab dependent cross-reactivity between peanut and deciduous trees is common, this is a problem when diagnosing peanut allergy [2,9]. There are now other promising diagnostic methods available, such as component resolved diagnostics (CRD) and basophil allergen threshold activation (CD-sens). CRD involves investigating the presence of antibodies to different peanut allergen proteins. Basophil activation is a functional test investigating basophil activation after exposure to an allergen and the threshold sensitivity is a function of reactivity and the affinity of the allergen to cell-bound IgE-ab which measures the degree of allergen sensitivity [19,20] (CD-sens). This method has been shown to correlate with allergen sensitivity in vivo measured by bronchial, nasal and skin test titration and with a positive or negative oral food challenge [1,16,17].

We have earlier reported a girl with mono-sensitization to Ara h 8 and a negative CD-sens to peanut, who passed two oral peanut challenges but reacted with anaphylaxis after eating 300 g of roasted peanuts. However, she is still eating and tolerates a handful of peanuts (~40 g) [21]. Mittag et colleagues have earlier showed that rAra 8-protein is not stable and degraded in the roasting process [10]. However it has recently been shown that rAra h 8 protein in both raw and roasted peanuts is stable in experiments that mimic human digestion [22]. In our study we have shown that rAra h 8 can activate basophils in children sensitized to Ara h 8. Therefore one might presume that a systemic allergic reaction can occur if a large amount of peanut is eaten over a short time period.

The amount of peanut protein Ara h 8 is low in peanut and 80–90% of the peanut allergens belong to the storage proteins, Ara h 1, 2 and 3 [23]. A recent report show that only 8 μg per 1 g (0.8%) roasted peanuts are Ara h 8 proteins but the Ara h 8, *in vitro*, has proteolytic stability to gastric and pancreatic degradation [22].

In our study all children but one were negative in CD-sens to peanut probable because of the low content of Ara h 8 in roasted peanut. The child with positive CD-sens had at time of the challenge, but not at inclusion, low levels of IgE-ab to rAra h 2 (0.4 kU_A_/L) which could be a plausible explanation for the positive CD-sens result.

rAra h 8 has earlier been shown to cause histamine release from basophils investigated with basophil histamin release test [10]. In contrast to our study, they observed systemic reactions in 40% of their adult patients after peanut challenge. The patients had IgE-ab to Ara h 8 and to birch pollen but many also had IgE-ab to peanut storage proteins, Ara h 1, Ara h 2 or Ara h 3, which might explain why several of them reacted systemically to peanuts. A plausible explanation why the children in our study did not react at challenge could be that they were only IgE-sensitized to rAra h 8, the amount of rAra h 8 is low in roasted peanut and the quantity of peanut eaten were too low to cause an allergic reaction [22]. A more speculative explanation could be a low valency, assuming that IgE-ab binding to Ara h 8 is induced by a cross-reactive allergen (Bet v 1) and it is likely that substantially fewer epitopes are exposed by Ara h 8 than by Bet v 1.

Three children in the present study were negative in CD-sens to rAra h 8. Two of them had very low levels of IgE-ab to rAra h 8 and also a very small IgE-ab fraction size. The third child had a higher level of IgE-ab to rAra h 8 and the basophils responded to the positive control. However, there was no reaction after allergen stimulation (peanut, rAra h 8 or rGly m 4) despite having IgE-ab to them.

In the present study six children reported subjective OAS at the peanut challenges indicating that Ara h 8 could activate mast cells in the oral mucosa before gastric degradation which is in line with a report from Dirks and colleges showing that peanut allergens are taken up directly from the oral cavity [24]. However, we did not find any association between OAS and the CD-sens values to rAra h 8 or to the levels of IgE-ab to the different peanut allergens (P = ns). We speculate that a higher amount of peanuts can induce more severe allergic symptoms in both children with and without OAS.

We also investigated CD-sens to rGly m 4, in order to compare it with CD-sens to Ara h 8. Previous reports have shown that birch pollen-allergic individuals IgE-sensitized to Gly m 4 report more severe symptoms after drinking soy milk during birch pollen season than individuals sensitized to Ara h 8 who have eaten peanuts [2,25,26]. The children in our study were selected for having IgE-ab to Ara h 8, but 19/20 also had IgE-ab to Gly m 4. However, only 11 were CD-sens positive to rGly m 4. We did not perform soy challenges but it would be of great interest to investigate if the children with positive CD-sens tor rGly m 4 would react at an oral challenge to soy.

All peanut challenges and CD-sens analyses were performed in the same medical centers, Sachs’ Children’s and Youth Hospital and Karolinska University Hospital, respectively, but the number of patients in this study is limited and therefore the results should be interpreted with caution. Another limitation was that the amount of peanuts eaten in this study was rather low (11.1 g) although in concordance with clinical evaluation of peanut allergy in Sweden. However, it would be of great importance to challenge with a higher amount of peanuts to elucidate if the patient can tolerate unrestricted amounts of peanut or not.

## Conclusions

In conclusion, positive CD-sens to rAra h 8 show that the Ara h 8 IgE-ab sensitized basophils can be activated by a Ara h 8 allergen and initiate an allergic inflammation. Hence, children sensitized only Ara h 8 but not to peanut storage proteins may be at risk for systemic allergic reaction when eating larger amounts of peanuts but most likely don’t have to fear smaller amounts.

## Methods

### Study design

Children recruited to our study participated in another study investigating oral tolerance to peanuts [2]. At inclusion all children (n =160, aged 5–18 years) had IgE-ab to peanut > 0.35 kU_A_/L. They were also IgE-sensitized to Ara h 8 and birch (>0.35 kU_A_/L) but not to Ara h 1, Ara h 2 and Ara h 3 (<0.35 kU_A_/L) at inclusion. Twenty children were randomly selected for the present study by inviting the first two children/families each week that came for oral peanut challenge. A blood sample was drawn for CD-sens analysis, IgE and IgE-ab measurements the same day and before the challenge. Clinical background data were collected from medical records, interviews and questionnaires. Inclusion criteria were IgE-sensitization as described above and avoidance of peanut. Exclusion criteria for oral peanut challenge were previous anaphylaxis grade II or III after exposure to peanuts, ongoing infection or allergic reactions to other foods or inhalant allergens. The study was approved by the ethics committee in Stockholm, Sweden (Dnr 2010/1331-31/3) and the parents provided written consent.

### Peanut challenge

An open peanut challenge was performed using pure roasted peanuts every 20 minutes in 4 steps: 100 mg, 1 g, 5 g and an additional 5 g to a total of 11.1 g. The challenge was negative if no objective allergic symptoms occurred during one hour after the challenge was completed. If oral allergy syndrome (OAS) occurred, i.e. local symptoms from the oral cavity without any other symptoms [27], and if the OAS symptoms disappeared spontaneously without medication the challenge was regarded as negative. If a challenge was positive, i.e. objective symptoms from the skin, gastrointestinal tract, respiratory tract and/or cardiovascular system occurred a double-blind placebo-controlled food challenge (DBPCFC) was planned to follow.

### Blood sampling

Blood samples were collected the same day and before the challenge and stored at +4°C for a maximum of 24 hours before CD-sens analyses. Serum was separated and stored at −20°C pending analyses.

### Basophil analyses

Basophils from 100 μL whole blood/test were used and stimulated with 100 μL allergen in decreasing concentrations giving a final volume of 200 μl/test [14,15] Desalted roasted peanut extract (final concentration 2.5-2500 ng/ml), recombinant rAra h 8 (final concentration 0.05-500 ng/mL) and rGly m 4 (final concentration 0.05-500 ng/mL) (Thermo Fisher Scientific, Uppsala, Sweden) were tested. Anti-FcεRI (Bühlmann Laboratories AG, Schönenbuch, Switzerland) and N-formyl-methionyl-leucyl-phenylalanin (Sigma Chemical Co, St.) were used as positive controls. Stimulated leucocytes were stained for CD63 and CD203c (Immunotech, Marseille, France). Cell surface expression of CD203c was used for identification of basophils and CD63 was used for detection of activated basophils. The basophils were counted in a Navios flow cytometer (Beckman Coulter, Inc., Fullerton, CA, USA).

The percentage of CD63-positive basophils in the control sample was below 2.5% and the cut-off determining a positive test was set to 5% i.e. twice the background.

### Definitions of CD-sens

Basophil allergen threshold sensitivity, CD-sens, was measured with a dose response curve, as the lowest allergen concentration giving 50% (LC_50_) of maximum up-regulation of CD63. CD-sens is defined as the inverted value for LC_50_ multiplied by 100 ((1/LC_50_)100) and was used to describe the patient’s allergen sensitivity [14,15]. The higher the CD-sens, the greater the patient’s allergen sensitivity.

### Serological analyses

IgE and IgE-ab to peanut, birch pollen and the allergen components rAra h 1, 2, 3, 8, 9, rBet v 1 and rGly m 4 were determined in serum with ImmunoCAP® (Thermo Fisher Scientific) according to the manufacturer’s instructions. A positive test was defined as an IgE-ab level ≥0.1 kU_A_/L. The ratio of IgE-ab of total IgE (IgE-ab/total IgE) was calculated and designated the “IgE-ab fraction”.

### Statistics

The material tested was not normally distributed and therefore non-parametric analyses were used. The results are presented as median and (range) unless otherwise stated. Spearman rank order correlation (r_s_) was used to assess the association between the challenge, CD-sens and IgE-ab levels. Wilcoxon rank sum test was used to assess differences in children with and without OAS and levels of CD-sens and IgE-abs. Significance was considered at a p-value of <0.05. Analyses were performed using IBM SPSS Statistic 20.0, Chicago, Ill, USA.

### Availability of supporting data

The data set supporting the results of this article is included within the article and its additional files.

## Abbreviations

CD: Cluster of differentiation
CD-sens: Basophil allergen threshold sensitivity
IgE- ab: IgE-antibody
LC50: Lowest concentration
LPT: Lipid transfer protein
OAS: Oral allergy syndrome
DBPCFC: Double-blind placebo-controlled food challenge
